# Diversity of *Ascodipteron* and Host-Associated Variation in Parasitism Among Hipposiderid Bats in Yunnan, China

**DOI:** 10.3390/insects17101004

**Published:** 2026-09-29

**Authors:** Yifan Deng, Lin Zhang, Rong Xiang, Ran Zhao, Fan Yang, Ying Li, Xiaobin Huang

**Affiliations:** 1 Yunnan Provincial Key Laboratory for Zoonosis Control and Prevention, Institute of Pathogens and Vectors, Dali University, Dali 671000, China; d18191647332@126.com (Y.D.); 13525787204@163.com (R.Z.); 13988381898@163.com (F.Y.); 2467640317@126.com (Y.L.); 2 Jilin Provincial Key Laboratory of Animal Resource and Ecological Security, Northeast Normal University, Changchun 130117, China; zhangl309@nenu.edu.cn; 3 State Key Laboratory of Pathogen and Biosecurity, Academy of Military Medical Sciences, No. 20 Dongdajie, Fengtai District, Beijing 100071, China; xiangrong102501@163.com

**Keywords:** *Ascodipteron*, bat flies, species diversity, DNA barcoding, host–parasite associations, body condition, neosomy, Yunnan

## Abstract

*Ascodipteron* bat flies are specialized parasites of bats, but their diversity and parasite ecology in China remain poorly understood. We surveyed hipposiderid bats at ten localities in Yunnan Province, China, combining morphological identification, mitochondrial cytochrome c oxidase subunit I (*COI*) barcoding, and ecological analyses. Among 226 bats, 58 were infested; 72 neosomic females were collected, and 14 representative specimens were sequenced. Morphology, *COI* genetic distances, Assemble Species by Automatic Partitioning (ASAP), and maximum-likelihood phylogenetic analysis supported three taxa: *A. phyllorhinae*, *A. sanmingense*, and an undescribed *Ascodipteron* sp. Within-taxon *COI* distances were 0.199–0.512%, whereas between-taxon distances were 6.303–12.118%. Overall prevalence was 25.66%, and prevalence and mean parasite abundance varied among host species. Evidence for host-species differences in parasitism depended strongly on the absence of infestation in *Hipposideros gentilis*. Estimated associations of body condition with infestation occurrence and parasite abundance were positive but uncertain, and no clear associations were detected for the site-level environmental variables examined. These findings expand current knowledge of *Ascodipteron* diversity and host-associated parasitism in Yunnan and provide an integrative basis for further studies of species identification, host use, and parasite ecology in this genus.

## Simple Summary

*Ascodipteron* is a highly specialized group of bat parasites. After attaching to a host, females lose their wings, legs, and other appendages and become embedded in the host skin, forming a neosome. However, the species diversity of this genus in China and its parasitic associations with different bat hosts remain poorly understood. In this study, we surveyed hipposiderid bats at ten localities in Yunnan Province, China. A total of 226 bats were examined, and 72 *Ascodipteron* specimens were collected. Based on morphological characteristics and genetic barcoding, three *Ascodipteron* species were identified, including two known species and one species that has not yet been formally named, indicating that *Ascodipteron* diversity in Yunnan may still be incompletely documented. Prevalence and parasite abundance also differed among bat species, and host species may be an important factor associated with natural patterns of parasitism. These findings expand current knowledge of *Ascodipteron* diversity and host-associated parasitism in China and provide a basis for further comparisons of host associations and parasite ecology among species.

## 1. Introduction

Bat flies are obligate blood-feeding dipteran ectoparasites of bats, and their species diversity has long been an important focus of research on bat ectoparasites [1]. Traditionally, bat flies have been classified primarily into the families Streblidae and Nycteribiidae; however, molecular phylogenetic studies have shown that the traditional Streblidae is not monophyletic, and its higher-level classification continues to be revised [2,3]. *Ascodipteron* represents one of the most highly specialized bat fly groups in terms of life history. After establishing on a host, females lose their wings, legs, and other appendages and become embedded in the host skin, forming a neosome [1,4]. Approximately 18 valid *Ascodipteron* species are currently recognized worldwide, and at least six species have been recorded or described from China, including *A. longiascus*, *A. phyllorhinae*, *A. sanmingense*, *A. speiserianum*, *A. guoliangi*, and *A. euryale* [4,5,6,7]. The continuing discovery of new species in recent years suggests that the species diversity of *Ascodipteron* in China may still be incompletely documented.

After neosomy, *Ascodipteron* females lose their wings, legs, and other appendages, and many external structures commonly used in dipteran taxonomy are consequently absent. Species-level identification therefore relies mainly on a limited set of morphological characters, including setation of the labial theca, localized cephalothoracic structures, and annular rows of abdominal setae [4]. However, closely related species may differ only subtly in these characters, and neosomic specimens are also prone to damage during collection and preservation, limiting the reliability of species identification based solely on external morphology. Recent taxonomic studies of *Ascodipteron* in China have increasingly integrated morphological evidence with mitochondrial molecular markers, including mitochondrial cytochrome b (*Cytb*) and cytochrome c oxidase subunit I (*COI*) sequences [5,6,7,8], indicating that molecular data have become an important complement to morphological identification in this genus. Nevertheless, existing molecular studies have focused mainly on the identification and description of individual taxa, and systematic comparisons of species composition and interspecific genetic differentiation among samples from different localities and hosts remain limited. Therefore, combining morphological identification with *COI* sequence analysis can help validate species assignments, compare genetic differentiation among taxa, and identify potentially undescribed species.

Compared with taxonomic research, the parasite ecology of *Ascodipteron* in natural host populations remains much less understood. Previous studies of bat flies have shown that parasitism may be associated with host species, body size, geographic range, population abundance, and roosting habits [9,10], whereas the effects of individual host characteristics such as sex, age, and body condition may vary among specific host–parasite systems [11,12]. Environmental factors, including temperature and precipitation, may also influence parasitism patterns through their effects on host distribution, roosting conditions, and parasite life-history processes [13,14]. However, current knowledge of *Ascodipteron* is still derived largely from taxonomic descriptions, host records, and attachment-site observations, and comparative studies involving multiple host species across multiple localities remain scarce [4,5,6]. Therefore, field surveys encompassing multiple hosts and localities are needed to further evaluate the relationships of host characteristics and environmental factors with *Ascodipteron* parasitism.

Yunnan Province has documented records of *Ascodipteron* [7], and hipposiderid bats represent an important host group for this genus in China [4,5,6]. However, systematic comparisons of *Ascodipteron* species composition, *COI* genetic differentiation, and parasitism across different hosts and localities in Yunnan remain lacking. In this study, we combined morphological identification with *COI* DNA barcoding to investigate the species composition and genetic differentiation of *Ascodipteron* associated with hipposiderid bats in Yunnan, and further evaluated, at the genus level, the relationships of host species, individual host characteristics, and site-level environmental factors with infestation occurrence and overall parasite abundance. Specifically, we addressed three questions: (1) which *Ascodipteron* species were present in the collected material, and whether morphological identification was concordant with *COI*-based analyses of representative specimens; (2) how *COI* haplotypes were distributed among host species and sampling localities; and (3) whether *Ascodipteron* infestation occurrence and overall parasite abundance were associated with host species, individual host characteristics, and site-level environmental factors.

## 2. Materials and Methods

### 2.1. Study Area, Bat Capture, and Bat Fly Collection

**Figure 1:**
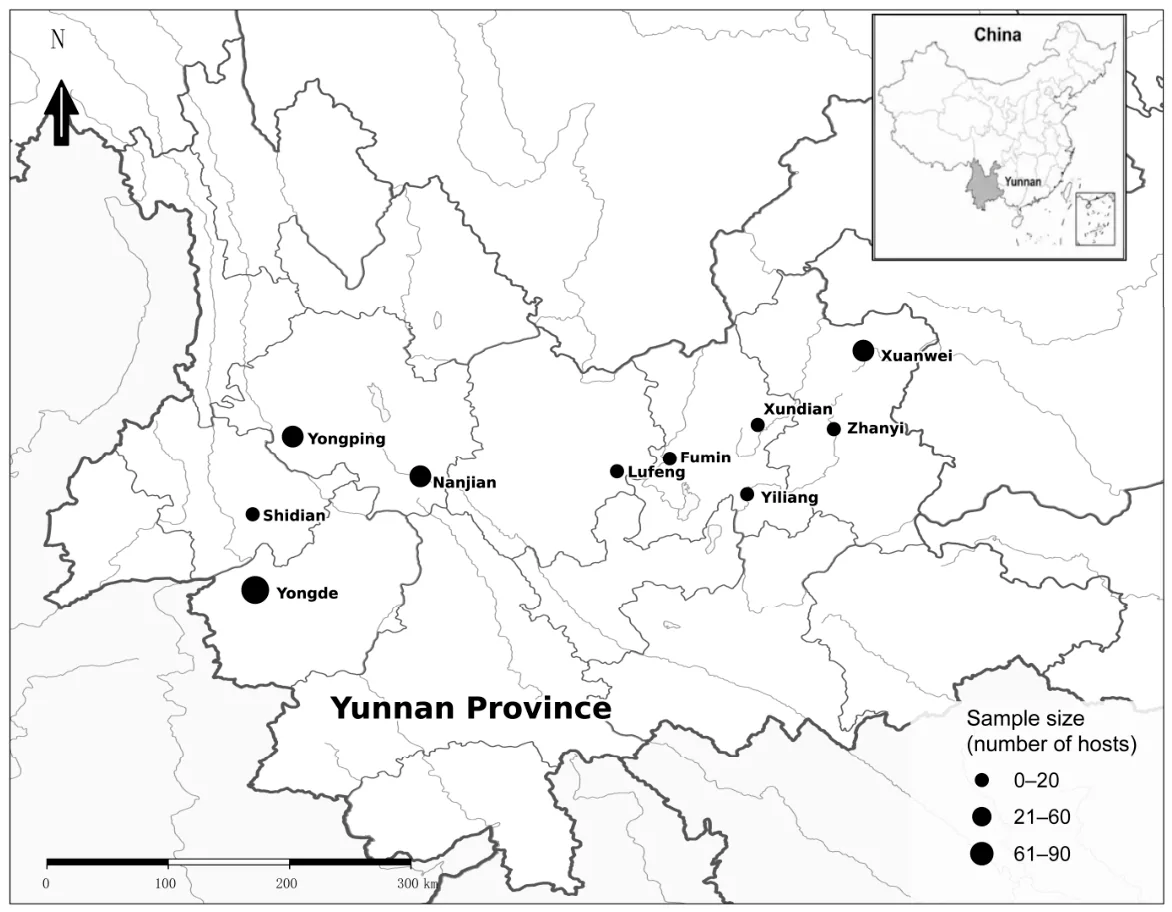
Geographic distribution of the ten bat sampling localities in Yunnan Province, China. Circle size represents the sample size category at each locality. The inset shows the location of Yunnan Province within China.

Field surveys were conducted from July to September 2025 at ten localities in Yunnan Province, China: Yongde, Shidian, Yongping, Nanjian, Lufeng, Fumin, Yiliang, Xundian, Zhanyi, and Xuanwei (Figure 1). At each locality, sampling was scheduled over two or three days from 18:00 to 24:00 each evening, giving a total scheduled sampling window of 12–18 h per locality. Mist nets were deployed at cave entrances and along nearby flight paths to capture hipposiderid bats. Host species were identified based on morphological characteristics and echolocation calls [15,16]. Sex was determined from external genital characteristics, and age class was assessed according to the degree of ossification and fusion of the phalangeal epiphyses; individuals with incompletely fused epiphyses were classified as non-adults, whereas those with fully fused epiphyses were classified as adults [17]. Each bat was subsequently examined at the bases of the ears, wing membranes, and other common attachment sites of *Ascodipteron*. Specimens embedded in the skin or subcutaneous tissue were carefully removed using sterile needles and forceps. Host forearm length was measured to the nearest 0.01 mm using a digital caliper (model 500-196-30; Mitutoyo Corporation, Kawasaki, Japan), and body mass was measured to the nearest 0.01 g using an electronic balance (model BSM220.4; Shanghai Zhuojing Electronic Technology Co., Ltd., Shanghai, China). After parasite collection and host measurements were completed, bats were released at their sites of capture. Individual bat flies were preserved separately in 1.5 mL microcentrifuge tubes containing 95% ethanol, and the corresponding host information and specimen identification numbers were recorded. Host species composition at each sampling locality is presented in Table 1. All bat capture, handling, and parasite collection procedures were conducted in accordance with a protocol approved by the Medical Ethics Committee of Dali University (MECDU-202104-27; approved on 18 April 2021).

### 2.2. Species Identification of Bat Flies and COI Sequence Analysis

All neosomic females were examined under a Leica DM500 microscope (Leica Microsystems, Wetzlar, Germany). Identification followed published descriptions [4,5,6,7], focusing on the number and distribution of labial-theca setae, folds or thickening of the lateral vertex, and annular rows of abdominal setae. Fourteen specimens were selected for *COI* sequencing based on morphology, host origin, and locality. Genomic DNA was extracted from the head and thorax of some specimens and from the whole body of others using a TIANamp Genomic DNA Kit (TIANGEN, Beijing, China). Voucher specimens are deposited at the Institute of Pathogens and Vectors, Dali University, China. *COI* was amplified using LCO1490/HCO2198 [18] and sequenced by Sangon Biotech (Shanghai, China). Reads were assembled and quality-checked in Geneious Prime v2026.1.2; BLAST (https://blast.ncbi.nlm.nih.gov/Blast.cgi, accessed on 26 September 2026) comparisons complemented morphological identification. Sequences were deposited in GenBank (PZ092934–PZ092947).

The newly generated *COI* sequences and *Ascodipteron* reference sequences retrieved from NCBI GenBank were aligned using ClustalW in MEGA 12 [19,20], manually checked, and trimmed. Uncorrected p-distances and ASAP v1.0 partitions [21] were evaluated alongside morphology. Maximum-likelihood phylogeny was reconstructed in MEGA 12 using the GTR + G + I model and 1000 bootstrap replicates, with *Glossina morsitans* (JQ246706) as outgroup. For haplotype analyses, *COI* sequences were trimmed separately to the region shared by all sequences within each taxon, yielding lengths of 552 bp for *Ascodipteron sanmingense*, 523 bp for *A. phyllorhinae*, and 665 bp for *Ascodipteron* sp. Median-joining haplotype networks were constructed separately for each taxon using the PopART v1.7 implementation [22] with ε = 0. Host and locality records were used descriptively, without inferring host specificity or geographic distribution patterns from the selected specimens.

### 2.3. Parasitism and Associated Factors

Individual bats were used as the statistical units in the parasitological analyses. Parasitism was described using prevalence (*P*), mean abundance (M_A_), and mean intensity (M_I_), calculated according to standard definitions in parasite ecology [23]. Infestation occurrence was defined as whether at least one *Ascodipteron* individual was detected on a host, whereas overall parasite abundance was defined as the number of *Ascodipteron* individuals recorded on each examined host, including zeros for uninfested hosts. Infestation occurrence and overall parasite abundance were used as the response variables in subsequent analyses of associated factors. Because parasite counts among infested hosts were concentrated at relatively low values, mean intensity was used only as a descriptive measure and was not analyzed separately using count regression. Host body condition was quantified using the scaled mass index (SMI), which adjusts body mass for structural body size [24]. For each host species, SMI was calculated as follows:

$${SMI}_{i}=M_{i}{\left(\frac{L_{0}}{L_{i}}\right)}^{b_{SMA}}$$

where *M_i_* is body mass, *L_i_* is forearm length, *L*_0_ is the mean forearm length within each species, and *b_SMA_* is the scaling exponent estimated from a standardized major axis regression of natural log-transformed body mass on natural log-transformed forearm length. The estimated *b_SMA_* values were 2.453 for *Aselliscus stoliczkanus*, 4.955 for *Hipposideros armiger*, 1.703 for *H. gentilis*, and 3.370 for *H. larvatus*. To account for differences in body size among host species, the calculated SMI values were subsequently Z-standardized within each species to a mean of 0 and a standard deviation of 1. Thus, a one-unit increase in standardized SMI represents an increase in one standard deviation relative to the mean SMI of the corresponding host species.

Environmental variables included the normalized difference vegetation index (NDVI), annual mean temperature (AMT), and annual precipitation (AP). The values of these variables at the ten sampling localities are presented in Table 2. AMT and AP were obtained from the BIO1 and BIO12 variables, respectively, in WorldClim v2.1 [25] and were used to characterize the long-term climatic background of each sampling locality. NDVI was calculated from MODIS vegetation-index data and averaged over the sampling period [26] to represent vegetation conditions during field sampling. Because all three environmental variables varied at the locality level, they were first Z-standardized across the ten sampling localities and then assigned to individual hosts according to sampling locality. These variables therefore represent site-level environmental variation rather than independent environmental measurements at the individual-host level. Pearson correlation coefficients and variance inflation factors (VIFs) were used to assess correlations and collinearity among environmental variables. To avoid including correlated environmental predictors in the same model, each candidate model contained no more than one environmental variable. In addition, a principal component analysis (PCA) was performed on standardized NDVI, AMT, and AP using the ten sampling localities as the units of analysis, and PC1 was used as an integrated environmental gradient in sensitivity analyses. Because the environmental variables comprised only ten independent locality-level observations, their relationships with parasitism were interpreted primarily as exploratory site-level associations.

Infestation occurrence was analyzed using a generalized linear model (GLM) with a binomial error distribution and logit link function, whereas overall parasite abundance was analyzed using a Poisson GLM with a log link function. Candidate predictors included host species, sex, age class, within-species standardized SMI, and one of NDVI, AMT, or AP. Only main effects were considered, with no interaction terms. Host species, sex, age class, and SMI were allowed to occur in all possible combinations, while each model contained at most one environmental variable. Together with the intercept-only model, this resulted in a set of 64 prespecified candidate models.

Candidate models were ranked according to the small-sample corrected Akaike information criterion (AICc), and Akaike weights (w_i_) were used to quantify the relative support for each model [27,28]. Models with ΔAICc < 2 were used only to describe candidate models receiving support similar to that of the best-supported model. A 95% cumulative Akaike-weight model set was constructed by ranking candidate models from the highest to the lowest Akaike weight, and full model averaging was performed across this model set. For models in which a given predictor was absent, the corresponding coefficient was set to zero during model averaging [27,28]. The summed Akaike weights of all candidate models containing a given predictor (Σw_i_) were also calculated to describe the model-inclusion support for that predictor. Because Σw_i_ is influenced by both the structure of the candidate model set and the frequency with which a predictor occurs among candidate models, it should not be interpreted as either an effect size or an absolute measure of variable importance [29]. Among the 64 candidate models, host species, sex, age class, and SMI each occurred in 32 models, whereas NDVI, AMT, and AP each occurred in 16 models. Therefore, Σw_i_ values for the four host- and individual-level predictors were compared under the same candidate-model structure, whereas Σw_i_ values for individual environmental predictors were treated descriptively and were not directly compared with those of the host- and individual-level predictors. Environmental variables were interpreted primarily on the basis of model-averaged effect estimates, 95% confidence intervals, and the results of sensitivity analyses.

For the multilevel categorical predictor host species, Σw_i_ represents the model-inclusion support for host species as an overall model term and therefore cannot be summarized by a single odds ratio (OR) or incidence rate ratio (IRR). In the binomial models, exponentiated regression coefficients for continuous predictors or individual levels of categorical predictors were expressed as ORs, whereas the corresponding coefficients in the Poisson models were expressed as IRRs. For both model types, 95% Wald confidence intervals and *p* values were reported.

Because none of the 23 *H. gentilis* individuals were infested with *Ascodipteron*, complete or near-complete separation could potentially occur in the binomial models. The primary analyses therefore first retained all four host species and were subsequently repeated after excluding *H. gentilis* to assess the sensitivity of the main results to the inclusion of this zero-infestation host species. In addition, the best-supported logistic model based on AICc fitted to the full dataset was reanalyzed using bias-reduced logistic regression as a sensitivity diagnostic for separation [30], rather than as a replacement for the primary multimodel inference. Model fit for the principal GLMs was assessed using simulated residual diagnostics implemented in DHARMa, with additional checks for overdispersion and zero inflation in the overall parasite-abundance models. Given that the environmental variables represented only ten independent locality-level observations and that host species were unevenly distributed among sampling localities, additional generalized linear mixed models (GLMMs) with sampling locality fitted as a random intercept were used as sensitivity analyses [31]. These analyses were used primarily to determine whether accounting for locality-level clustering altered the direction of the SMI effect and were not considered to fully resolve confounding between host composition and spatial sampling structure.

All statistical analyses were conducted in R version 4.6.0. Data management was performed primarily using the dplyr package; GLMs were fitted using functions in the base stats package; AICc-based model selection and model averaging were conducted using MuMIn; simulated residual diagnostics were performed using DHARMa; supplementary analyses of separation in logistic regression were conducted using brglm2; and GLMMs with sampling locality as a random intercept were fitted using lme4.

## 3. Results

### 3.1. Morphological Identification and COI Analyses Identified Three Ascodipteron Species

A total of 226 hipposiderid bats were examined, from which 72 neosomic *Ascodipteron* females were collected. Based on morphological identification of all specimens, together with molecular verification using *COI* sequences from 14 representative individuals (all showing sequence similarities > 97%), three *Ascodipteron* species were identified: *A. phyllorhinae* (42 individuals), *A. sanmingense* (23 individuals), and an undescribed *Ascodipteron* sp. (7 individuals). *Ascodipteron phyllorhinae* and *A. sanmingense* were generally similar in external morphology but differed clearly in the labial theca and lateral vertex. In *A. phyllorhinae*, the dorsal surface of the labial theca bears approximately 25–30 pale, peg-like spiniform setae, and the lateral vertex lacks conspicuous longitudinal folds or thickening. In contrast, *A. sanmingense* bears more than 50 normally pigmented, peg-like spiniform setae on the dorsal surface of the labial theca, and the lateral vertex exhibits conspicuous longitudinal folds or thickening. Both species possess complete annular rows of abdominal setae (R1–R5). In specimens of *Ascodipteron* sp., the arrangement of the abdominal setal rows R1–R5 could not be clearly resolved. The small number of specimens available, together with damage to some individuals, also limited reliable assessment of other diagnostic morphological characters. Although *COI* sequences distinguished these specimens from the two named species, the available morphological evidence was insufficient for a reliable species-level identification. They were therefore provisionally retained as *Ascodipteron* sp., pending clarification of their taxonomic status.

**Figure 2:**
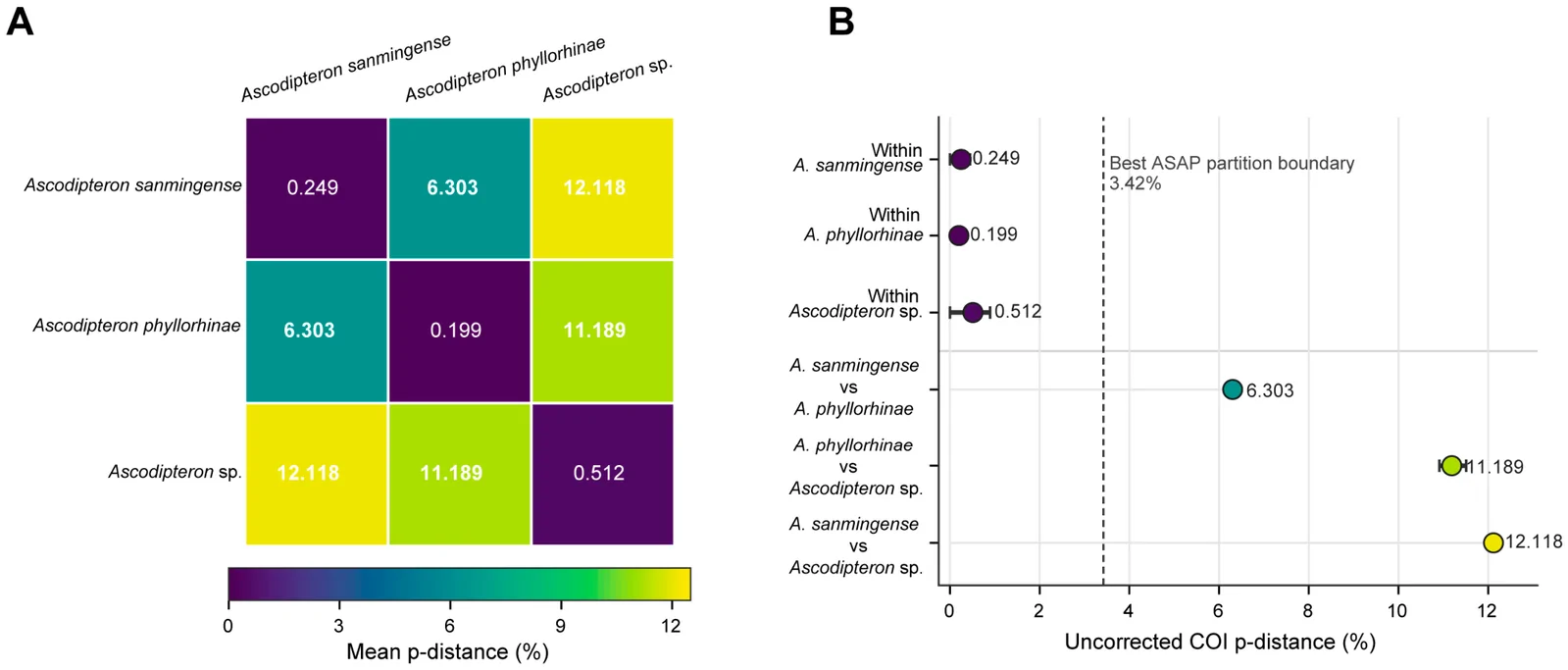
*COI* genetic differentiation within and among three *Ascodipteron* species. (**A**) Matrix of mean uncorrected *COI* p-distances within and between species. (**B**) Mean uncorrected *COI* p-distances within and between species; horizontal bars indicate the observed minimum–maximum range of pairwise distances. The dashed vertical line indicates the distance boundary (3.42%) associated with the best-ranked ASAP partition and should not be interpreted as a universal species-delimitation threshold.

Mean uncorrected *COI* p-distances within each of the three species were low, at 0.199% for *A. phyllorhinae*, 0.249% for *A. sanmingense*, and 0.512% for *Ascodipteron* sp. In contrast, interspecific genetic distances were substantially greater: 6.303% between *A. sanmingense* and *A. phyllorhinae*, 11.189% between *A. phyllorhinae* and *Ascodipteron* sp., and 12.118% between *A. sanmingense* and *Ascodipteron* sp. Mean genetic distances among the three species were all greater than the distance boundary associated with the best-ranked ASAP partition (Figure 2). The best-ranked ASAP partition likewise divided the study sequences into three groups, consistent with the groupings indicated by morphological identification and *COI* genetic distances.

**Figure 3:**
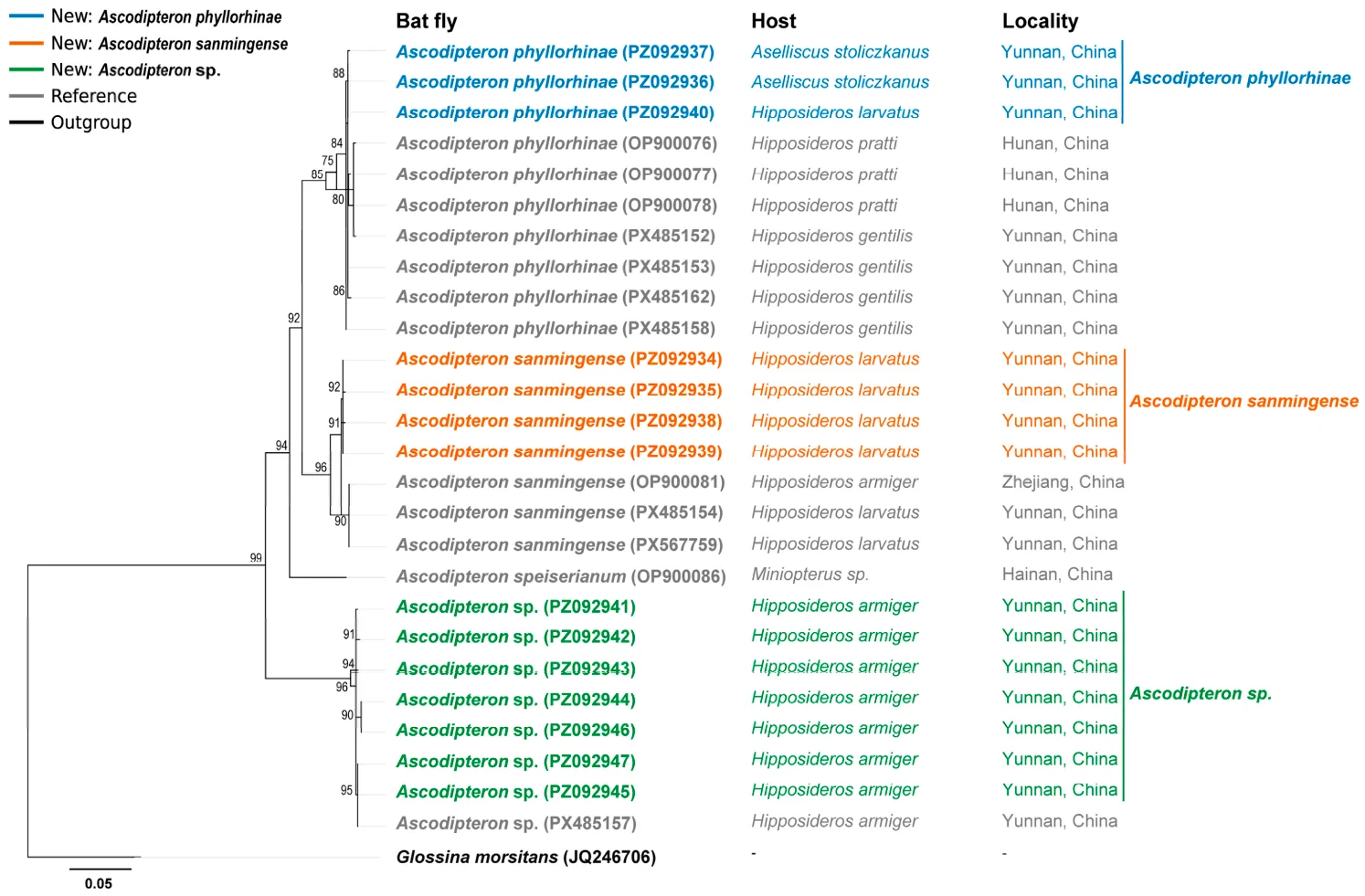
Maximum-likelihood phylogeny of *Ascodipteron* based on mitochondrial *COI* sequences. Newly generated sequences are colored according to the three *Ascodipteron* species, with host species, sampling locality, and GenBank accession number indicated in each tip label; reference sequences are shown in gray. Node values indicate bootstrap support (%) based on 1000 replicates, and the scale bar represents nucleotide substitutions per site.

The three newly generated *A. phyllorhinae COI* sequences clustered with published reference sequences of this species, whereas the four *A. sanmingense* sequences formed a distinct cluster with publicly available reference sequences of *A. sanmingense*. The remaining seven sequences clustered with an unidentified *Ascodipteron* reference sequence to form another distinct lineage and showed substantial genetic differentiation from the two named species (Figure 3). Overall, morphological identification, *COI* genetic distances, ASAP, and maximum-likelihood phylogenetic analysis yielded concordant groupings of the three species.

**Figure 4:**
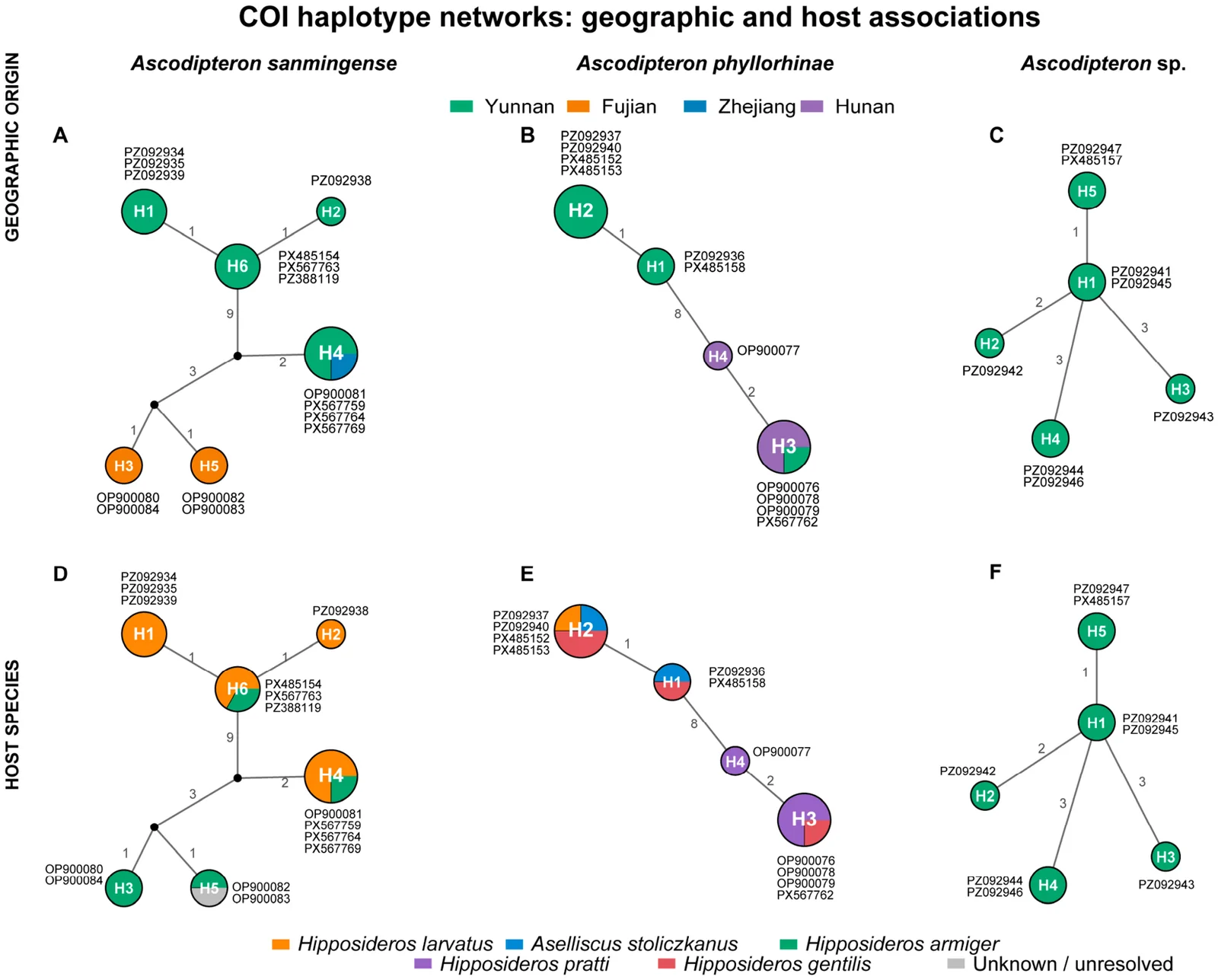
Median-joining haplotype networks based on newly generated *COI* sequences and GenBank reference sequences. (**A**,**D**) *Ascodipteron sanmingense*; (**B**,**E**) *A. phyllorhinae*; (**C**,**F**) *Ascodipteron* sp. The upper (**A**–**C**) and lower (**D**–**F**) panels show the same networks colored by geographic origin and host species, respectively. Circle size is proportional to haplotype frequency, and pie sectors indicate the proportions of sequences from each origin. Sequence accession numbers are listed beside the circles. Haplotypes are numbered independently within each taxon. Numbers on connecting lines indicate mutational steps, and small black nodes represent inferred intermediate haplotypes. Gray indicates unknown or unresolved host identity.

Haplotype analysis identified six, four, and five *COI* haplotypes in *A. sanmingense*, *A. phyllorhinae*, and *Ascodipteron* sp., respectively (Figure 4). The networks of the two named species each comprised two haplotype groups separated by longer mutational paths, whereas the remaining haplotypes of *Ascodipteron* sp. differed from the central haplotype by only 1–3 mutational steps. Haplotype sharing across provinces and across host species was observed in both named species.

### 3.2. Ascodipteron Parasitism Among Host Species

**Figure 5:**
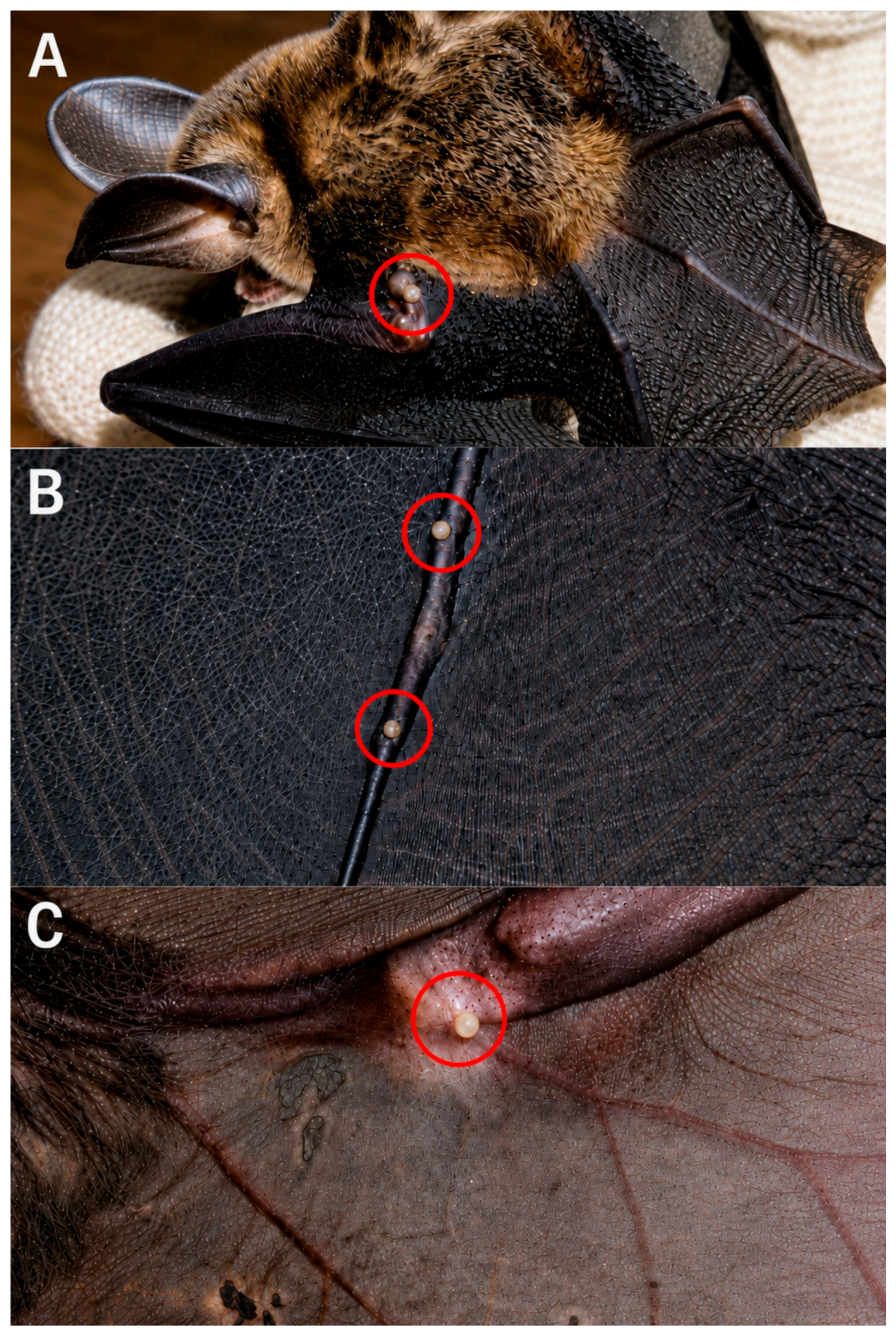
*Ascodipteron* and their hipposiderid bat hosts. (**A**) *Aselliscus stoliczkanus*; (**B**) *Hipposideros armiger*; (**C**) *Hipposideros larvatus*. Red circles indicate neosomic *Ascodipteron* females embedded in host tissue.

The overall prevalence of *Ascodipteron* infestation was 25.66%. Prevalence and mean abundance were highest in *Hipposideros larvatus*, followed by *H. armiger* and *Aselliscus stoliczkanus*; no infestation was detected in the examined *H. gentilis* (Table 3). Figure 5 documents females embedded in host tissue under field conditions.

### 3.3. Associations of Host Species and SMI with Ascodipteron Parasitism

At the locality level, AMT was strongly positively correlated with AP (*r* = 0.742), whereas correlations of NDVI with AMT (*r* = 0.278) and AP (*r* = −0.070) were weak; all three variables had VIF values below 3. In the PCA, PC1 explained 59.04% of the environmental variation and was driven mainly by AMT and AP, with absolute loadings of 0.716 and 0.670, respectively, whereas the absolute loading of NDVI was 0.197. The Pearson dispersion parameter for the overall parasite-abundance model was 0.954, and DHARMa simulated-residual diagnostics showed no clear evidence of overdispersion or zero inflation. The Poisson GLM was therefore retained as the primary model for parasite abundance.

**Figure 6:**
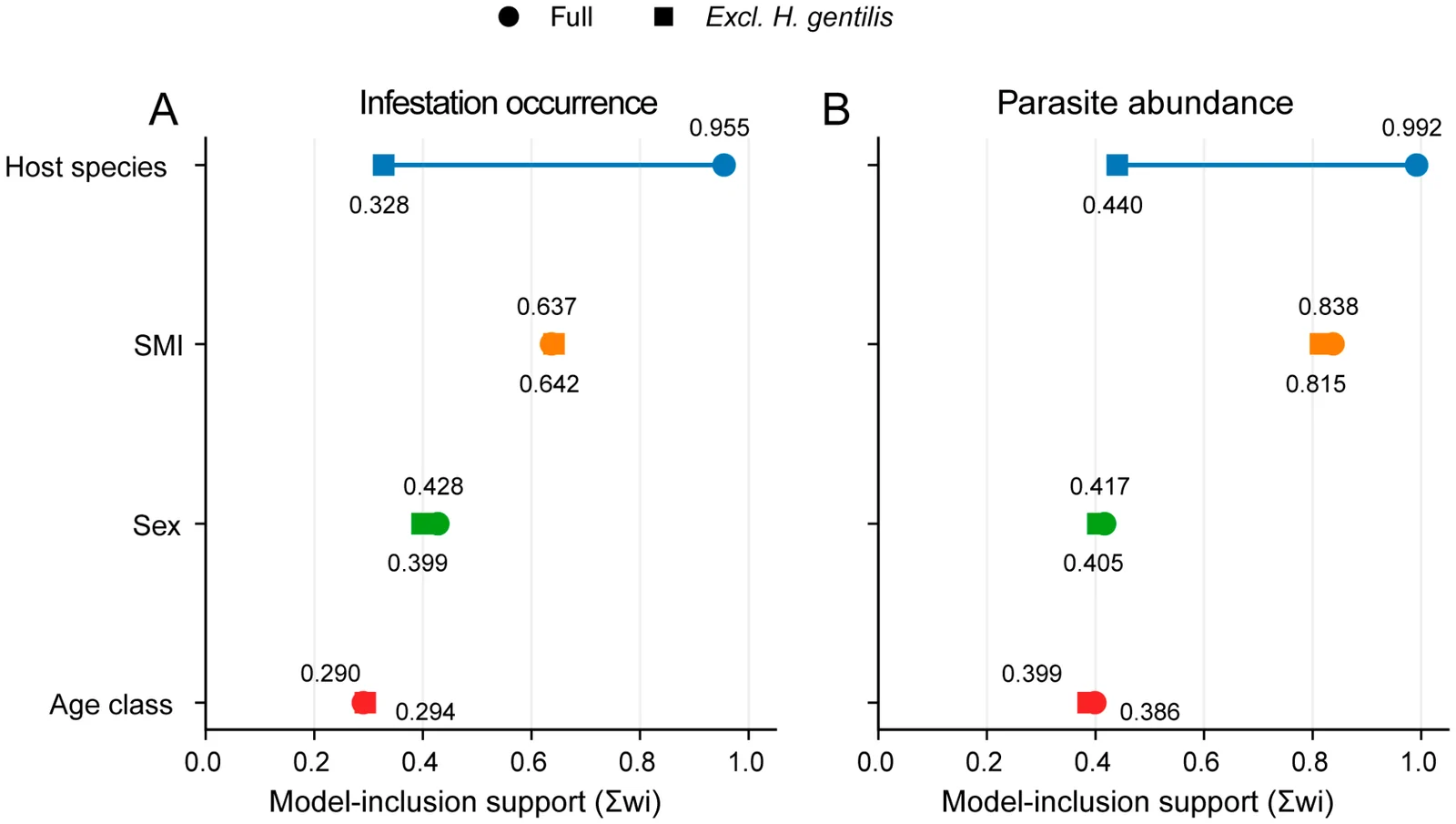
Model-inclusion support of host- and individual-level predictors for *Ascodipteron* infestation occurrence (**A**) and parasite abundance (**B**). Circles represent the complete four-host dataset (Full), and squares represent analyses excluding the zero-infestation host species *Hipposideros gentilis* (Excl. *H. gentilis*). Connected points represent the same predictor across datasets, and numerical labels indicate summed Akaike weights (Σw_i_). Host species, SMI, sex, and age class occurred with equal frequency in the candidate model set and are therefore directly comparable.

**Figure 7:**
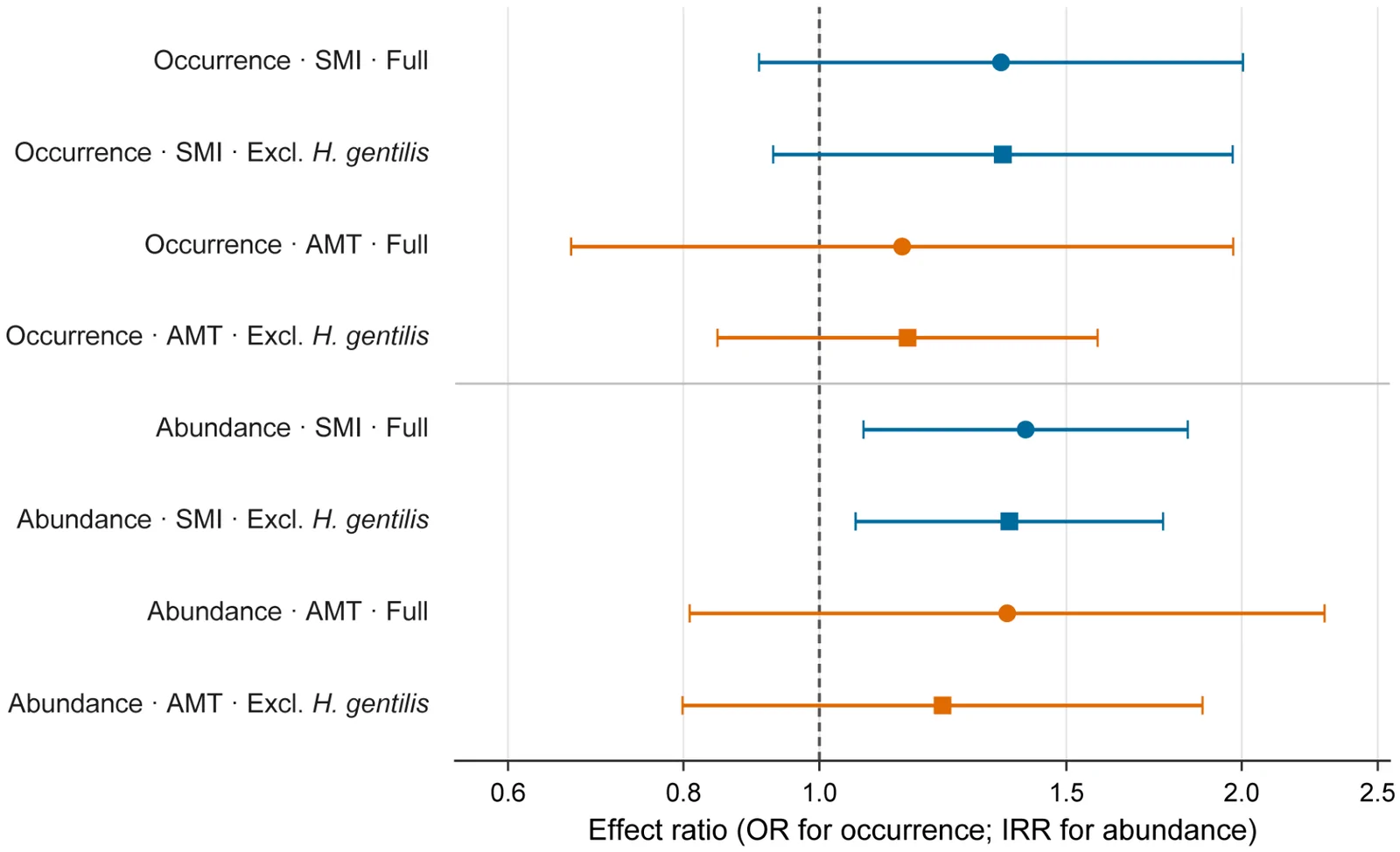
Full model-averaged effects of SMI and AMT on *Ascodipteron* infestation occurrence and parasite abundance. Effect estimates are expressed as odds ratios (ORs) for infestation occurrence and incidence rate ratios (IRRs) for parasite abundance. Circles represent the complete four-host dataset (Full), and squares represent analyses excluding *Hipposideros gentilis* (Excl. *H. gentilis*). Points indicate model-averaged effect estimates, horizontal bars indicate 95% Wald confidence intervals, and the dashed vertical line denotes the null effect (OR/IRR = 1).

For infestation occurrence, the best-supported model based on AICc in the full dataset included host species, SMI, and AMT, and 13 candidate models had ΔAICc < 2. Among the four directly comparable host- and individual-level predictors, host species received the highest model-inclusion support (Σw_i_ = 0.955), followed by SMI (0.637), sex (0.428), and age class (0.290) (Figure 6A). After excluding the zero-infestation host species *H. gentilis*, seven models had ΔAICc < 2. Model-inclusion support for host species decreased to Σw_i_ = 0.328, whereas support for SMI remained similar and became the highest among the host- and individual-level predictors (Σw_i_ = 0.642; Figure 6A). Model-averaged effect estimates for SMI were positive in both datasets, but the 95% confidence intervals included the null value of 1 (Full: OR = 1.228, 95% CI: 0.810–1.860, *p* = 0.333; Excl. *H. gentilis*: OR = 1.225, 95% CI: 0.820–1.830, *p* = 0.321). AMT likewise showed no clear association (Table 4; Figure 7). When the best-supported logistic model based on AICc for the full dataset was reanalyzed using bias-reduced logistic regression, the estimated effect of SMI increased (OR = 1.418, 95% CI: 0.999–2.013, *p* = 0.050), but the confidence interval still included 1; this result was therefore treated only as a sensitivity diagnostic for complete or near-complete separation. A similar pattern was observed for overall parasite abundance. In the full dataset, the best-supported model based on AICc again included host species, SMI, and AMT, and six candidate models had ΔAICc < 2. Host species received the highest model-inclusion support (Σw_i_ = 0.992), followed by SMI (0.838), sex (0.417), and age class (0.399) (Figure 6B). After excluding *H. gentilis*, ten candidate models had ΔAICc < 2. Model-inclusion support for host species decreased to Σw_i_ = 0.440, whereas SMI retained relatively high support (Σw_i_ = 0.815) and became the highest-supported host- or individual-level predictor (Figure 6B). Model-averaged effect estimates for SMI were positive in both datasets (Full: IRR = 1.314, 95% CI: 0.947–1.823, *p* = 0.102; Excl. *H. gentilis*: IRR = 1.282, 95% CI: 0.936–1.755, *p* = 0.122), but the 95% confidence intervals included 1. AMT likewise showed no clear association (Table 4; Figure 7).

Replacing the individual environmental variables with the integrated environmental PC1 produced broadly similar results. In the full dataset, model-inclusion support for host species was Σw_i_ = 0.974 for infestation occurrence and 0.995 for overall parasite abundance, whereas the corresponding values for SMI were 0.665 and 0.834. In the overall parasite-abundance analysis, the estimated effect of SMI remained positive (IRR = 1.304, 95% CI: 0.947–1.796, *p* = 0.104), whereas Env_PC1 showed no clear association (IRR = 1.234, 95% CI: 0.717–2.127, *p* = 0.448). In additional GLMMs with sampling locality included as a random intercept, the direction of the SMI effect remained positive: OR = 1.396 (95% CI: 0.981–1.986, *p* = 0.064) for infestation occurrence and IRR = 1.364 (95% CI: 1.062–1.753, *p* = 0.015) for overall parasite abundance. In the overall parasite-abundance model that also included AMT, the estimated effect of SMI was IRR = 1.492 (95% CI: 1.137–1.958, *p* = 0.004), whereas that of AMT was IRR = 1.541 (95% CI: 0.969–2.450, *p* = 0.068). However, these GLMMs showed singular fits, with the variance of the locality-level random intercept equal to or close to zero. These results were therefore treated only as sensitivity analyses accounting for locality-level clustering and were not considered independent inferential evidence.

Overall, host species received high model-inclusion support in the full dataset, but this result was sensitive to the inclusion of the zero-infestation host species *H. gentilis*. In contrast, the estimated effects of SMI were consistently positive across the primary and sensitivity analyses, although the 95% confidence intervals from the primary model-averaged analyses included the null value. No clear associations were detected for the site-level environmental variables.

## 4. Discussion

This study identified three *Ascodipteron* taxa in hipposiderid bats from Yunnan: *A. phyllorhinae*, *A. sanmingense*, and an undescribed *Ascodipteron* sp. Morphological and *COI* evidence supported their differentiation, while parasitological analyses revealed host-associated variation at the genus level. However, support for host species was sensitive to the inclusion of the uninfested *H. gentilis*. Body-condition estimates were consistently positive but uncertain, and no clear associations were detected for the measured environmental variables.

The agreement between morphological identification and *COI* analyses supports their complementary use in distinguishing closely related *Ascodipteron* taxa, consistent with recent taxonomic studies of the genus [5,6,7]. The detection of a morphologically and genetically distinct *Ascodipteron* sp. suggests that regional diversity remains incompletely characterized. Nevertheless, limited specimens, partial damage, and reliance on mitochondrial *COI* prevent a definitive assessment of its taxonomic status, and we therefore refrain from formally describing it here. The expanded haplotype analysis adds information on variation within the recognized taxa. In both named species, some haplotypes were shared across host species and provinces, showing that differentiation among taxa coexisted with shared *COI* variation across host and geographic records within taxa. This distinction is relevant because taxonomic separation does not itself establish ecological host specialization. Conversely, shared haplotypes document common sequence variants but do not establish the frequency of host switching or contemporary gene flow. The networks therefore complement the taxonomic analyses without resolving population structure, particularly given representative sampling, uneven reference coverage, and different sequence lengths among taxa.

The host-associated differences in parasitism broadly agree with studies linking bat-fly infestation to host ecology, including roosting habits, body size, distribution, and abundance [9,10]. However, the decline in host-species support after excluding *H. gentilis* indicates that the full-dataset result was strongly influenced by the contrast between this uninfested host and the other species. Its absence of infestation deserves particular caution because the reference dataset contains *A. phyllorhinae* sequences recorded from *H. gentilis* (Figure 3 and Figure 4). Thus, the present finding cannot be interpreted as general host incompatibility. Ecologically, differences in roost use and contact with local parasite populations could alter exposure. Biologically, grooming or immune defense could influence parasite establishment, although neither was measured. Methodologically, the sample of 23 *H. gentilis* and restricted sampling period cannot exclude low or temporally variable infestation. These possibilities cannot be distinguished with the current data. More generally, Singaporean nycteribiid host associations were interpreted partly in relation to host availability and single-species roosting [12], while mitochondrial and nuclear data from Kenya supported a broad host range in *Penicillidia fulvida*, with *COI* structure more readily explained by geography than host family [32]. Together, these comparisons show why observed host associations must be interpreted within their ecological and spatial context. In our survey, uneven host representation among sites and the pooling of *Ascodipteron* taxa mean that host identity, exposure, and parasite composition remain difficult to separate.

The positive SMI estimates suggest a possible relationship between host condition and parasitism, but the primary model-averaged confidence intervals included the null value. The direction agrees with the tendency for *A. speiserianum* to occur on *Miniopterus fuliginosus* in moderate to better condition in Taiwan [33]. In contrast, Péter et al. found negative body-condition associations for nycteribiid flies on European *M. schreibersii*, alongside positive associations for wing mites [34]. Work from Singapore also indicates that associations involving host condition, sex, and age depend on the particular host–parasite combination [12]. These findings suggest that a single expectation for the condition–parasitism relationship is unlikely to apply across parasite groups and host contexts. Our positive estimates are compatible with greater resource availability on hosts in better condition, but do not demonstrate preferential infestation of such hosts or exclude differences in exposure and defense. Moreover, SMI is an indirect index derived from body mass and forearm length [24]. Concurrent measurements of condition and parasitism cannot distinguish effects of host condition on infestation from consequences of infestation or shared seasonal influences.

The lack of clear environmental associations should likewise be interpreted in relation to sampling scale and predictor choice. Pilosof et al. detected climatic associations with bat-fly abundance across 43 localities in Venezuela, while responses to anthropogenic disturbance differed among host species [13]. Our analyses instead relied on ten independent locality-level observations, limiting the information available to assess environmental relationships. AMT and AP represented long-term regional climate, whereas NDVI reflected vegetation during sampling; none directly measured conditions within roosts. The association of bat-fly parasitism with roost permanence and enclosure reported by Patterson et al. [9] suggests that relevant variation may occur below the resolution of these predictors. Accordingly, the present results leave local environmental effects unresolved and should not be interpreted as evidence that environmental conditions are unimportant.

Several limitations define the next steps needed to evaluate these interpretations. Only 14 of the 72 collected flies were sequenced, while ecological analyses pooled *Ascodipteron* taxa; species-specific associations could therefore be obscured by differences in parasite composition. Uneven host representation among localities also restricted comparisons of the same hosts across sites, and sampling from July to September did not cover the annual cycle. Further work should increase individual parasite identification and use a crossed sampling design encompassing the same host species at multiple sites and multiple host species within sites. Better-preserved specimens and nuclear or multilocus data would strengthen taxonomic assessment, while seasonal sampling, direct roost measurements, and physiological indicators such as fat reserves or immune function would help distinguish host condition, exposure, and environmental contributions to parasitism.

## 5. Conclusions

This study provides evidence of *COI* differentiation and host-associated variation in *Ascodipteron* parasitism in Yunnan. The taxonomic status of *Ascodipteron* sp. remains unresolved. Host-associated differences in parasitism were sensitive to the inclusion of the uninfested *H. gentilis* sample and could not be fully separated from the uneven distribution of hosts among localities. Body-condition associations were positive but uncertain, and no clear associations were detected for the measured site-level environmental variables. Further taxonomic assessment and balanced sampling of host species across localities are needed to clarify species-specific host associations and the factors underlying these patterns.

## Figures and Tables

**Table 1:** Host species composition across the ten sampling localities in Yunnan Province, China. Numbers in parentheses indicate the total number of bats examined at each locality.

Site	*Hipposideros armiger*	*Hipposideros larvatus*	*Hipposideros gentilis*	*Aselliscus stoliczkanus*
Yongde (89)		89		
Shidian (4)	4			
Zhanyi (13)	13			
Xuanwei (34)	34			
Fumin (18)			18	
Yiliang (5)			2	3
Xundian (3)	2		1	
Yongping (21)	21			
Nanjian (26)	5			21
Lufeng (13)	10	1	2	
Total (226)	89	90	23	24

**Table 2:** Environmental variables across the ten sampling localities in Yunnan Province, China.

Site	AP (mm)	AMT (°C)	NDVI
Yongde	1284	19.68	0.64
Shidian	1127	17.08	0.41
Zhanyi	992	15.02	0.25
Xuanwei	1005	13.70	0.22
Fumin	852	16.15	0.47
Yiliang	932	14.97	0.35
Xundian	875	14.52	0.60
Yongping	987	17.05	0.30
Nanjian	914	15.21	0.65
Lufeng	865	15.37	0.70

**Table 3:** Host-specific parasitological metrics for genus-level *Ascodipteron* parasitism across four hipposiderid bat species. Prevalence confidence intervals are Wilson 95% confidence intervals.

Host Species	Examined Hosts	Infested Hosts	Collected Flies	Prevalence (%)	Wilson 95% CI (%)	Mean Abundance	Mean Intensity
*Hipposideros larvatus*	90	32	38	35.56	26.44–45.85	0.422	1.188
*Hipposideros armiger*	89	22	30	24.72	16.93–34.60	0.337	1.364
*Aselliscus stoliczkanus*	24	4	4	16.67	6.68–35.85	0.167	1.000
*Hipposideros gentilis*	23	0	0	0.00	0.00–14.31	0.000	—

**Table 4:** Full model-averaged effects and model-inclusion support for SMI and AMT for *Ascodipteron* infestation occurrence and parasite abundance.

Response	Dataset	Predictor	Σw_i_	Effect Ratio	95% Wald CI	*p* Value
Infestation occurrence	Full	SMI	0.637	OR = 1.228	0.810–1.860	0.333
Infestation occurrence	Full	AMT	0.247	OR = 1.130	0.669–1.908	0.648
Infestation occurrence	Excl. *H. gentilis*	SMI	0.642	OR = 1.225	0.820–1.830	0.321
Infestation occurrence	Excl. *H. gentilis*	AMT	0.382	OR = 1.129	0.766–1.663	0.540
Parasite abundance	Full	SMI	0.838	IRR = 1.314	0.947–1.823	0.102
Parasite abundance	Full	AMT	0.467	IRR = 1.227	0.724–2.078	0.447
Parasite abundance	Excl. *H. gentilis*	SMI	0.815	IRR = 1.282	0.936–1.755	0.122
Parasite abundance	Excl. *H. gentilis*	AMT	0.424	IRR = 1.132	0.758–1.690	0.544

Note: Σw_i_ denotes the summed Akaike weights of candidate models containing the corresponding predictor. Host species, sex, age class, and SMI each occurred in 32 candidate models, whereas NDVI, AMT, and AP each occurred in 16 models. Therefore, Σw_i_ values for individual environmental variables are reported descriptively and are not directly compared with those of host- and individual-level predictors. Effect estimates were obtained by full model averaging within the 95% cumulative Akaike-weight model set. Host species is a multilevel categorical predictor and cannot be summarized by a single OR or IRR.

## Data Availability

The *COI* sequences generated in this study have been deposited in the NCBI GenBank database under accession numbers PZ092934–PZ092947. The remaining data supporting the findings of this study are available from the corresponding author upon reasonable request.

## Abbreviations

The following abbreviations are used in this manuscript:

AICc	Small-sample corrected Akaike information criterion
AMT	Annual mean temperature
AP	Annual precipitation
ASAP	Assemble Species by Automatic Partitioning
*COI*	Cytochrome *c* oxidase subunit I
*Cytb*	Cytochrome *b*
GLM	Generalized linear model
GLMM	Generalized linear mixed model
IRR	Incidence rate ratio
NDVI	Normalized difference vegetation index
OR	Odds ratio
PCA	Principal component analysis
PC1	First principal component
SMI	Scaled mass index
VIF	Variance inflation factor
